# Demystifying upper limb hybrid prostheses—a scoping review

**DOI:** 10.3389/fresc.2025.1610336

**Published:** 2025-11-17

**Authors:** Sam Walters, Elena Seminati, Benjamin Metcalfe, Nicola Y. Bailey, Elise Catherine Pegg

**Affiliations:** 1 Department of Mechanical Engineering, University of Bath, Bath, United Kingdom; 2 Department for Health, University of Bath, Bath, United Kingdom; 3 Centre for the Analysis of Motion, Entertainment Research Applications, University of Bath, Bath, United Kingdom; 4 Bath Institute for the Augmented Human, University of Bath, Bath, United Kingdom; 5 Department of Engineering, King’s College London, London, United Kingdom; 6 School of Engineering, Newcastle University, Newcastle, United Kingdom

**Keywords:** prosthesis, hybrid, body-power, myoelectric, review

## Abstract

**Introduction:**

Hybrid-power is a prosthesis class that combines body-power and external-power into a singular embodiment. The class is rarely discussed in literature and is ill-defined, with the term “hybrid” being used to describe a broad range of upper-limb prostheses. This is despite the increased use of hybrid-power prostheses in clinical practice for treating people with above-elbow amputations; there is also little literature assessing their performance relative to the functional benchmarks of body-power or external-power prostheses. This scoping review aims to identify the various subcategories of hybrid prosthesis that exist, with an explicit focus on hybrid-power devices, and to report on the designs and use-cases of hybrid-power devices presented in clinical and research contexts. Where possible, comparisons are made between the performance of hybrid-power devices and other active prostheses.

**Methodology:**

This study follows PRISMA 2020 systematic review reporting guidelines to identify, sort, and select relevant literature from databases. Searches were conducted on three research databases (Scopus, Web of Science, PubMed) and two patent databases (eSpacenet, Derwent Innovations Index) to identify relevant sources on the topic of hybrid-powered prostheses. 142 unique research papers were identified from the three identified research databases, which were screened by title and abstract and further filtered following a full text review, leaving 13 relevant studies and 2 patents which underwent full-text screenings by the lead-author.

**Results:**

Five prominent categories of “hybridisation” were identified: hybrid-power, hybrid-control, hybrid-strategy, hybrid-actuation, and hybrid-feedback. Within the hybrid-power class, two prominent use-cases were identified: increasing active control inputs and reducing the physical effort necessary to operate a prosthesis. Additional use-cases were found within research, including increasing the number of grasps available for a transradial prosthesis, and providing flexible control options in areas with limited resources.

**Discussion:**

Insufficient quantitative evidence was found to draw any conclusions about the performance of hybrid-power prostheses relative to body-power or external-power devices. Further research should be conducted into the testing of conventional hybrid-power devices using standard clinical means, to establish a meaningful benchmark performance that future developments in research can draw from.

## 1 Introduction

Hybrid-power prostheses are a class of upper limb prosthesis combining the two most common active prosthesis classes of body-power and external-power. Active prostheses allow the user to directly control the movement of the prosthesis without external manipulation. The body-power and external-power classes remain the primary focus of prosthetic research, with comparisons being made between their respective merits (1–3). However, hybrid-power prostheses remain notably absent from such discussions. The conversation is perhaps muddied by the fact the term “hybrid” is used to describe prostheses in multiple contexts, beyond the conventionally attributed definition of being the combination of body-power and external-power. A discussion of hybrid-powered prostheses necessitates a clear understanding of the other upper-limb prosthetic classes and what distinguishes them from one another.

### 1.1 Prosthesis classes

**Figure 1:**
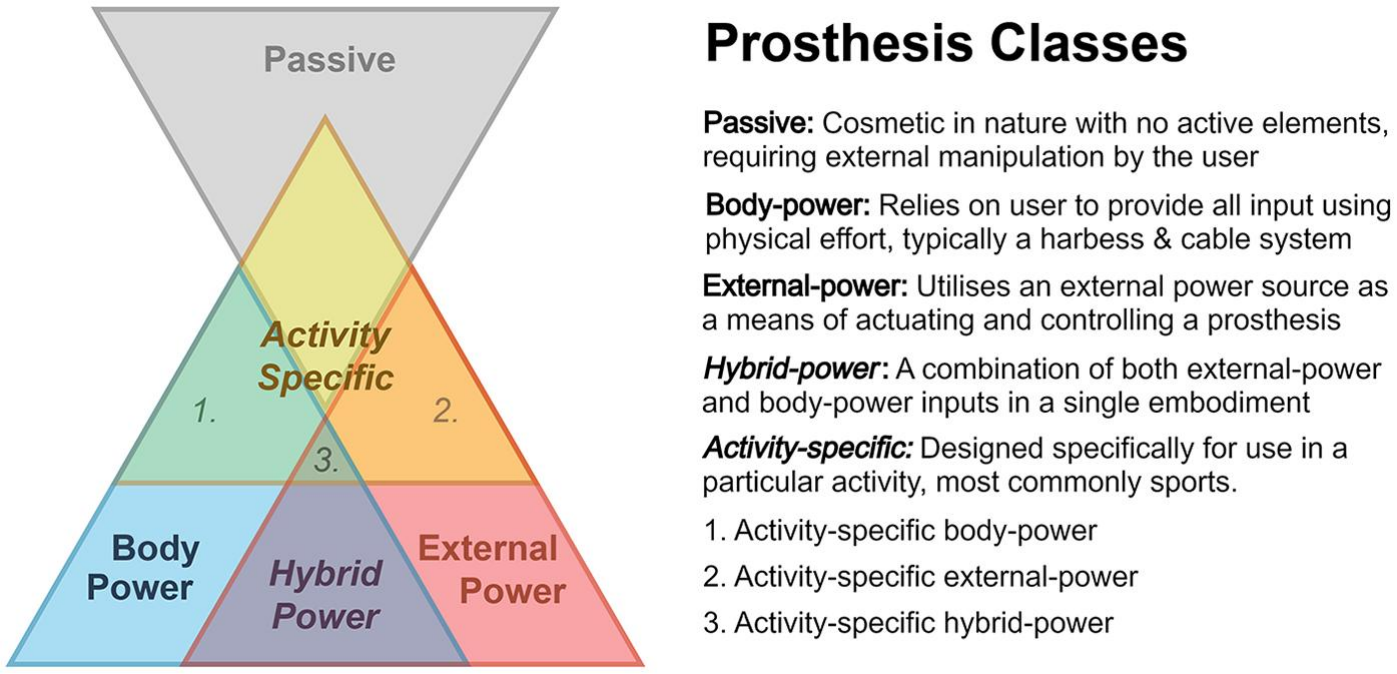
Upper-limb prosthetic classes. The five commonly accepted classes are labelled as such, namely passive, body-power, external-power, hybrid-power, and activity specific. The size of each section is not representative of the popularity of each class.

Figure 1 presents the five upper-limb prosthetic classes. There are three primary classes: passive, body-power and external-power, and two sub-classes existing exclusively within the intersection of these three classes: hybrid-power and activity-specific. There are a further three tertiary classes that exist as a conceptual consequence of how secondary and primary classes overlap with one-another, these being activity-specific variations of body-power, external-power and hybrid-power.

#### 1.1.1 Passive

A static prosthesis resembling the contralateral limb as closely as possible, typically prescribed to restore a sense of cosmesis to the patient. Unlike body-power and external-power, the passive class has no means of active control, and as such is functionally limited to being a static support for the intact hand (4).

#### 1.1.2 Activity-specific

Specialised prostheses designed to hold particular geometries or replicate certain tool, such as a terminal device for gripping handlebars or pliers for fine technical work, respectively (5). The majority of examples are passive, although a few commercial options offer means of active control for high intensity sports, such as a body-powered activity-specific prosthesis allowing the user to plant ski poles for Nordic skiing (6).

#### 1.1.3 Body-power

Exploits the physical effort of the user to exert active control over a joint or set of joints. This is traditionally embodied by a shoulder harness that uses the relative displacement between the arm and trunk to operate a control cable, which flexes a split-hook and/or elbow joint. Other means of embodying body-power have been explored in literature, such as harnessing the medium of hydraulics/pneumatics (7) or even using breathe control (8), however, the breadth of body-power design variations are minimal relative to the domain of external-power (9).

#### 1.1.4 External-power

Uses a source of power independent from the patient to create movement, using sensors and actuators to create a link between user input and the actuator output. The class of external power almost exclusively refers to electrical power, although the means of actuation in these embodiments may vary significantly. The most common embodiment, however, is a bionic hand actuated using brushless DC (direct-current) motors and operated with control signals collected from surface electromyography (sEMG) sensors placed over a range of muscle groups. These sensors detect the electrical excitement (flexion) of muscles, which may be used to operate the various degrees of freedom of the prosthesis (10, 11).

#### 1.1.5 Hybrid-power

**Figure 2:**
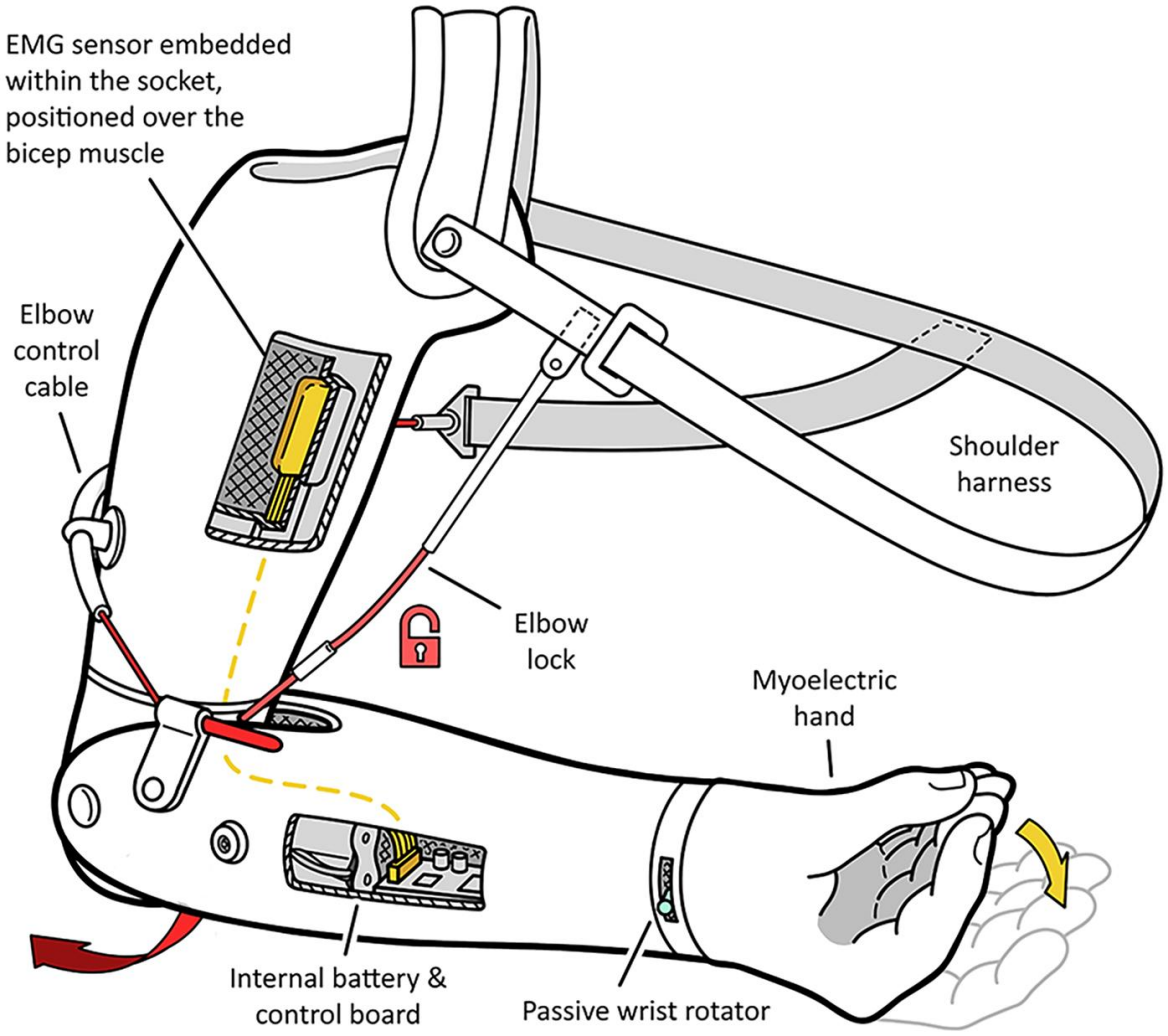
Example of a transhumeral hybrid prosthesis, featuring a body-powered elbow and locking mechanism, a passive wrist rotator, and an electric hand controlled using sEMG sensors in the socket. Electronics are conventionally stored within the shell of the lower arm.

The combination of two disparate means of powered control into a singular embodiment, which in current practice refers almost exclusively to the pairing of body-power and external-power. Consequently, the combination of passive components and active components of a single class is not considered a “hybrid-power” prosthesis, as the passive components are by definition not powered. Hybrid-power prostheses are traditionally prescribed to patients with above-elbow limb absence (12), the most common embodiment being an electric hand with a body-powered elbow, as shown in Figure 2, or an electric elbow with a body-powered split-hook. Unlike body-power and electric prostheses, hybrid-power prostheses are not manufactured commercially. Instead, they are an emergent consequence of “limb banks” (13): stocked components that clinicians have access to that may be mixed and matched according to the patients' needs. The prosthetist may take elements from one class to compensate for the deficits of the other, although good knowledge of prostheses is required to combine the two classes harmoniously (14). For instance, body-powered components may provide improved control and reliability, whereas externally-powered components may enhance versatility and grip strength. The choice of components the clinician has available to combine is highly dependent, however, on the resources the clinician has access to, which can vary significantly between countries, and even between neighbouring clinics.

### 1.2 The hybrid-power gap

The prescription rate of hybrid-power prostheses in clinical practice is unclear, due to their relative lack of documentation. McFarland et al. indicate a prescription rate of 20% for above-elbow amputees, accounting for approximately 10% of total upper-limb prosthesis prescriptions (12). The survey population of a study by Wanamaker et al. aligns with this population estimate, with 4 of the 17 participants with above-elbow amputations being prescribed a hybrid prosthesis (∼23.5%) (15). Although there are clinicians that regularly adopt hybrid-power prostheses in clinical practice, few if any clinical guidelines exist regarding their use and prescription. As such, their prescription typically relies on the individual experience and testimony of the clinicians who adopt them (13, 16), with little in the way of research into their functional benefits compared to other prostheses. This may be a consequence of their unstandardised designs, being the culmination of multiple disparate components, making quantification and generalisations of their designs and use-cases more difficult than other active classes to define.

Their continued use in clinical practice, especially for patients with bilateral above-elbow amputations (13, 16), highlights the need for an investigation into patient outcomes when prescribed these devices. In addition, a closer examination of which prosthetic units are chosen when constructing a hybrid-power prosthesis and what use-cases the various embodiments present is needed, to document successes and failures in previous cases to assist clinicians with future prescriptions and help inform their choices. To the authors knowledge, there are no existing academic sources that engage in a thorough discussion of the benefits and use-cases of hybrid-power prostheses. Of the studies that do mention their use, they are often forced to share the same category as myoelectric prostheses (12), despite their significant differences, and limiting the individual knowledge that can be drawn from them.

There is an additional need to establish the differences between these contexts of the term in research contexts to dispel confusion in their discussion and make categorisation of the different categories easier. By identifying the discussed gaps in knowledge, this scoping review will highlight areas for future research, which will be reported upon in the discussion.

### 1.3 Aims and objectives

Given the limited and heterogeneous literature on hybrid-power prostheses, this study was conducted as a scoping review with the aim of characterising the variations of hybrid-power prosthesis embodiments that exist in literature, and to identify the conditions under which they are prescribed. In the process of identifying relevant literature, examples of the range of applications the term “hybrid” is attributed to are grouped into multiple subcategories to reduce uncertainty around the term. Where relevant, outcome measures used to assess the functional performance and qualities of hybrid-power embodiments are identified and compared against other embodiments where possible. Finally, gaps within the literature of hybrid-power devices are identified and commented upon, and recommendations for future research to fill these gaps are made.

## 2 Methods

This review was conducted in accordance with the PRISMA 2020 review reporting guidelines (17).

### 2.1 Search strategy

Three research databases (Scopus, Web of Science, PubMed) and two patent databases (eSpacenet, Derwent Innovations Index) were searched for relevant sources, on 18/10/2024. The search strategy for all databases were identical, with the case for Scopus used as an example. The search criteria were based on terms pertaining to prostheses, specifically those pertaining to the upper limb, necessitating the inclusion of upper-limb elements such as “hand”, “elbow”, “wrist”, etc. This was to eliminate lower-limb prosthesis results. The search necessitated the explicit inclusion of the term “hybrid”, and references to disparate fields were excluded, which included “implant”, “tooth” and “hip”. The explicit search strategy for Scopus was designed as such: TITLE (prosthetic OR prosthesis OR prostheses) AND TITLE-ABS [hybrid AND (arm OR shoulder OR elbow OR hand OR finger OR wrist)] AND NOT (hip OR tooth OR implant) AND [LIMIT-TO (LANGUAGE, “English”)]. This strategy was adapted for all other databases. The search criteria were made deliberately open to explore the full range of contexts in which the term “hybrid” is used to define prostheses to clearly differentiate them and establish the boundaries between them. No publication date limit was imposed on the search criteria.

### 2.2 Study selection

**Figure 3:**
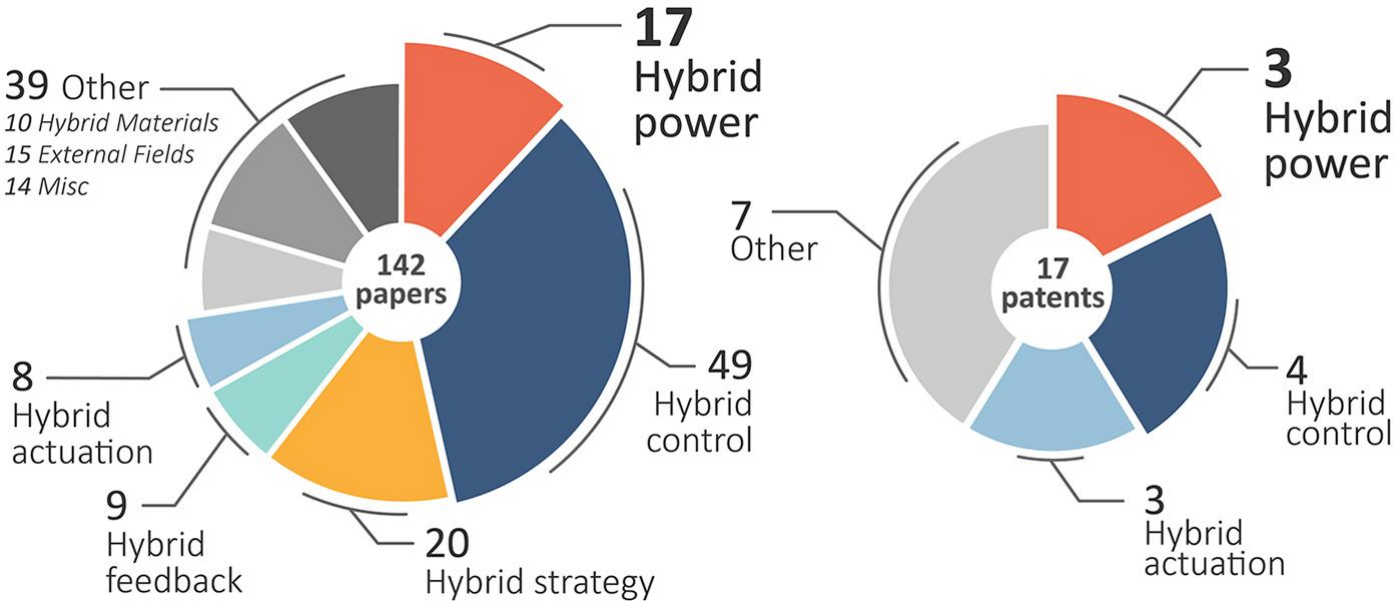
Distribution of categories captured in the search criteria.

Captured studies were gathered in a Covidence database, where duplicates were removed both automatically and manually. Two researchers conducted screenings of the captured title and abstracts: ECP and SW. Where conflicts arose, reviewers met to discuss and if agreement still could not be reached, a third reviewer was consulted to make the decision (ES). Cohen Kappa was used to measure the interobserver agreement between the two reviewers (18). Review stages progressed from title and abstract review to full paper review as described in Figure 3. The two patent databases were individually assessed by SW.

Inclusion criteria for research studies were as follows. The study must include an explicit description of the hybrid-power prosthesis being designed and/or studied, at least including a description of the terminal device and elbow joint (if applicable) and how they are respectively operated, in sufficient detail to be replicated. The study must also be available in English, or otherwise translated from the native publication language. No minimal number of participants was established for this scoping review. It is well understood amongst researchers in the field of prosthetics that sample sizes are typically small and underpowered. This is due to the population of people with upper-limb absence being relatively sparse, making the conduction of in-person studies especially difficult over questionnaires or surveys.

Studies were excluded if they were a review study, or an abstract with no full-text available. Studies were also excluded if the collected hybrid-power data was mixed with other prosthesis data, such that the hybrid data could not be analysed individually. Finally, studies that had not been peer-reviewed were excluded. The inclusion criteria for patents were the same as for the research literature, except for the peer-review requirement which is not relevant for patents. Initial title and abstract screening were observed, followed by full text review following the same approach.

Whilst a traditional means of quality assessment such as Downs and Black Checklist (19) was considered, there was a high heterogeneity in the study design, with some including human participants, while others explored solely design concepts. Of the studies that used human participants, the sample populations were small with highly varied outcome measures, to the extent that the employment of established assessment tools would offer little in the way of insight. As such, a custom set of criteria was employed that better reflects the contents of the studies in question, to assess author confidence in the findings of each study. The confidence criteria include:

**C1: Study design**: Does the study adhere to the recognised study designs and procedures outlined by Burns et al. levels of evidence (20)?
**C2: Aims**: Have the aims of the study been clearly established and are realistically achievable?
**C3: Participants**: Does the study use participants with upper-limb difference to test the hybrid-power prostheses discussed in the work?
**C4: Data collection**: Are standardised and replicable means of testing used to collect data that may be directly compared with other studies?
**C5: Data analysis**: Has the analysis of collected data been clearly described and conducted?
**C6: Conclusions**: Do the conclusions drawn in the study align with the results achieved?

These criteria were drawn and condensed from the guidelines established by Kmet et al. (21) for assessment of qualitative research. These broader criteria are applied to studies presenting both qualitative and quantitative research. As outlined by Kmet et al., ratings will be applied on a 0–2 scale, 0 being *No*, 1 being *Partial*, and 2 being *Yes*.

### 2.3 Data extraction

Data extraction from the included studies was conducted by one researcher (SW) knowledgeable in hybrid-power prostheses. Descriptions of the terminal device, wrist unit, elbow joint, and power class were extracted. Performance outcomes of these hybrid-power devices were also extracted, in addition to performance outcomes of other prosthetic devices which the hybrid-power devices were being compared to.

## 3 Results

The initial search found a total of 253 studies and 22 patents. 101 studies were automatically flagged as duplicates by Covidence and excluded, and an additional 10 duplicate studies and 5 patents were excluded manually. The remaining 142 studies and 17 patents were sorted into subcategories of “hybrid”, these categories being developed and iterated upon as screening progressed.

Only studies and patents pertaining to hybrid-power prostheses were included, resulting in 123 studies and 9 patents being excluded during the initial title and abstract screening. An absolute agreement of 93.0% was observed between the two reviewers in addition to a Cohen Kappa of 0.722, suggesting a moderate and acceptable level of agreement (22). A further 6 studies and 6 patents were excluded following full-text screening, with absolute agreement of 89.5% and a Cohen Kappa of 0.732. The revised distribution of the hybrid categories is shown in Figure 3. Note that several studies and patents did pertain to hybrid-power prostheses, however, did not meet all inclusion criteria.

**Figure 4:**
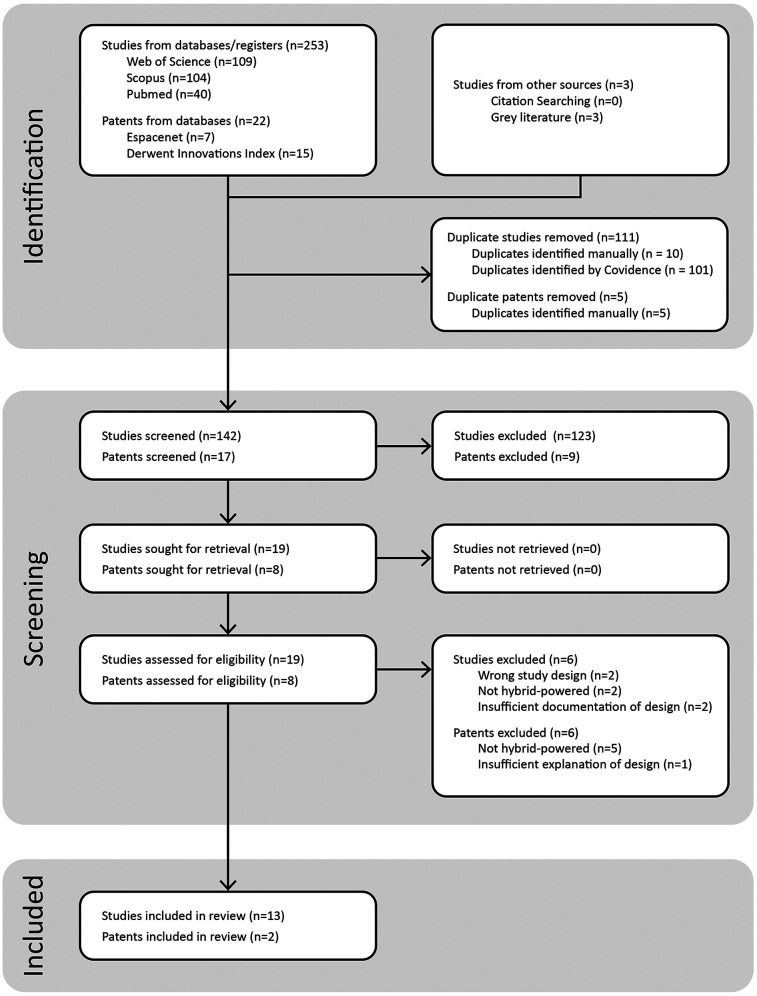
Systematic flowchart showing the filtering of database results and extraction of relevant studies and patents.

Thirteen relevant studies on upper-limb hybrid-prostheses remained, as well as two patents of the same. A flowchart of the process is outlined in Figure 4, illustrating the reasoning used at each stage for excluding studies and patents.

Following a full-text review of the remaining studies and patents, ­thorough data extraction was conducted in which relevant parameters were identified and recorded, including a description of the hybrid-power prosthesis design, the study design, the number of participants included (if applicable), and the outcome measures of the study. A similar extraction process was conducted for the two identified patents excluding any testing measures. As such, the only extracted data was a description of the hybrid-power prosthesis design, how it functioned, and what the identified novelty of the design was.

### 3.1 Categories of “hybrid” prostheses

During screening, five categories emerged for the term “hybrid”, as shown in Figure 3. The most prevalent was not hybrid-power, but *hybrid-control*, with more than four times the number of articles. In 1992 Childress established the differences between hybrid-power and hybrid-control (16), as follows:

- **Hybrid-power**: The use of body-power and external inputs in one prosthetic system, and the definition generally attributed to the term “hybrid prosthesis” (23–35).
- **Hybrid-control**: The use of two or more distinct external inputs that dictate the output of a prosthesis. Examples include the combination of EMG control and computer-vision (36, 37), EEG signals (38–40), or even other body parts (41). Madusanka et al. review of hybrid control systems provides a comprehensive review on this category (42).

This review identified three additional categories referred to with the “hybrid” prefix in literature:

- **Hybrid-actuation**: The use of two or more means of actuation, such as DC motors alongside shape memory alloys (43–45), or pneumatic actuators with DC motors (46).
- **Hybrid-strategy**: The use of joint/multiple control strategies for the control of electronic hands, typically employed to decipher sEMG signals for the control of multiple degrees of freedom (47–49).
- **Hybrid-feedback**: The use of two or more different stimuli or sensations to provide the user with haptic-feedback to restore a degree of proprioception (50, 51).

These categories describe significant differences in design implementation and use-cases, and as such may overlap with one another depending on the prosthesis embodiment. However, no such overlap exists within the captured set of studies on hybrid-power prostheses. Articles included in Figure 3 are grouped according to the most prominent category presented in the work, and the 17 works pertaining to hybrid-power prostheses are not all included in the review. This is due to aforementioned exclusion factors including study design, and insufficient information on the hybrid-power systems being investigated.

### 3.2 Use-cases of hybrid-power prostheses

The extracted data from the included studies and patents of this review are shown in Tables 1, 2, respectively. Of the 13 studies examining hybrid-power prostheses, there are thirteen unique instances. Three of the studies discuss the development of the SoftHand Pro (30–32), which will be considered as a singular instance. These thirteen may be categorised as conventional, or novel instances. Conventional in this context refers to a hybrid-power prosthesis created using pre-existing commercial components of varying power-classes, whereas novel refers to hybrid-power prostheses created with custom components or significant modifications made to commercial components.

**Table 1:** The table records general information from the relevant studies extracted in this review, details on the hybrid-power prosthesis design, and study results if applicable. Descriptions of the acronyms are as follows.

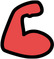 Body-power; 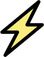 External power, 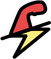 Joint body and external power, 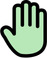 Passive							
Study	Limb level	Terminal device	Wrist unit	Elbow joint	No. of participants	Av. Age ± SD (yrs)	Outcome measures
Childress and Billock (23)	TH	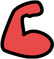	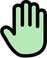	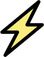	3	38 ± 4	Timed trials of manipulation tasks
Brenner and Brenner (24)	WD; TH; TH	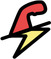 ; 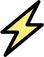 ; 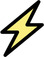	NR; NR; NR	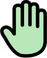 ; 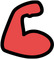 ; 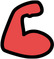	5^a^	47 ± 9	Iterative fitting, patient feedback
Bouwsema et al. (25)	TH	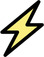	NR	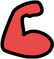	6	46 ± 11	Measurement of velocity profiles
Ricardo et al. (26)	TH	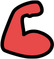	NR	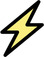	1	51 ± 0	DASH questionnaire, VAS, 400-points test
Law and Hewson (27)	TH	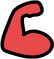	NR	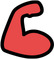 + 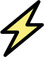	N/A	N/A	N/A
Thyberg and Johansen (28)	TH	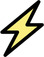	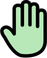	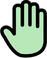 + 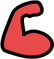	1	20 ± 0	Grip function test, patient feedback
Spaeth (29)	ED	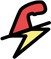	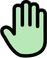	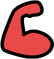	1	12 ± 0	Iterative fitting, patient feedback
Piazza et al. (30)	TR	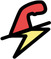	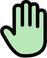	N/A	1	29 ± 0	Force measurements, Cybathlon 2016 trials
Godfrey et al. (31)	TR	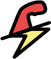	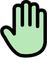	N/A	13^b^	34 ± 8	ACMC, SUS, BBT
Capsi-Morales et al. (32)	TR	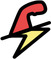	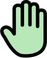	N/A	2	32 ± 9	ACMC, SUS, Cybathlon 2020 trial recreations
Polhemus et al. (33)	TR	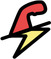	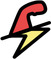	N/A	N/A	N/A	N/A
Semasinghe et al. (34)	TR	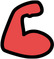 + 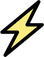	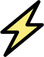	N/A	N/A	N/A	Kinematic Evaluation, Grasping analysis, Force measurements, Energy Consumption
Reboli et al. (35)	TH	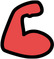 OR 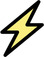	N/A	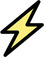	N/A	N/A	Measurement of circuit and motor delay

PH, partial hand; WD, wrist disarticulation; TR, transradial; TH, transhumeral; NR, not recorded; DASH, disabilities of the arm, shoulder and hand; VAS, visual analogue scale; ACMC, assessment of capacity for myoelectric control; SUS, system usability scale; BBT, box & blocks test; SD, standard deviation.

a Only 3 of the patients are prescribed hybrid-power prosthesis.

b 10 of the 13 participants were able-bodied and used a bypass prosthesis.

**Table 2:** Data of the two patents discovered that fulfil the Scoping Review criteria. Information on acronyms and icon meanings may be found in the caption of Table 1. Each patent falls into the International Patent Classification of A61F, denoting prostheses and other orthopaedic devices.

Patent	Patent ID	Assignees	Limb level	Terminal Device	Wrist Unit	Elbow joint
Seo-2023: Passive and active combined type prosthetic hand (57)	KR20230 085854-A	Seo K, Mo Y, Jae-Wan K, et al.	PH	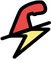	N/A	N/A
Zuniga-2020: UPPER LIMB PROSTHESES (58)	WO2020 081914-A1	Univ Nebraska, Zuniga J, Peck J, et al.	TR	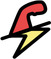	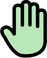	N/A

The confidence ratings of the respective studies are presented in Table 3. The spread of study types was relatively small, with only four types being represented, all being a low level of evidence of IV or lower, according to the levels of evidence of Burns et al. (20). The most represented study type was case reports, making up five of the thirteen studies, followed by four design proposals, three cross-sectional studies, and one case series. Whilst design proposals are not formally recognized by *Burns* et al. (20), Eshraghi et al. assign them as a category representing the sixth level of evidence, titled “Design and Development” when describing the most cited papers in prosthetic literature (52). The authors describe this group as “*design and development of prosthetic devices without clinical analysis on humans*”. As such, their level of evidence is denoted as “VI”, and all with the exception of Semasinghe et al. achieved only a low level of confidence. The two studies achieving the highest level of confidence were both cross-sectional studies, conducted by Bouwsema et al. (25) and Godfrey et al. (31).

**Table 3:** Confidence Ratings of studies included in this review. All studies are ranked according to six criteria (Cn), individually assessed by two assessors (SW & ECP) and disagreements resolved through discussion. Criteria are rated on a 0–2 scale, 0 being No, 1 being Partial, and 2 being Yes.

Study	Study design	Level of evidence	C1	C2	C3	C4	C5	C6	Confidence score	Confidence outcome
Childress and Billock (23)	Cross-sectional Study	IV	2	1	2	1	1	1	0.67	Medium
Brenner and Brenner (24)	Case-series	V	1	1	2	0	0	1	0.42	Medium
Bouwsema et al. (25)	Cross-sectional Study	IV	2	2	2	2	2	2	1	High
Ricardo et al. (26)	Case report	V	1	1	2	2	1	1	0.67	Medium
Law and Hewson (27)	Design & Development	VI	0	0	0	0	0	0	0	Low
Thyberg and Johansen (28)	Case report	V	1	0	2	0	1	1	0.42	Medium
Spaeth (29)	Case report	V	1	0	2	0	0	0	0.25	Low
Piazza et al. (30)	Case report	V	1	1	2	1	2	1	0.67	Medium
Godfrey et al. (31)	Cross-sectional Study	IV	2	2	2	2	2	2	1	High
Capsi-Morales et al. (32)	Case report	V	1	1	2	2	1	1	0.67	Medium
Polhemus et al. (33)	Design & Development	VI	0	1	0	0	0	0	0.08	Low
Semasinghe et al. (34)	Design & Development	VI	0	1	0	1	**2**	1	0.42	Medium
Reboli et al. (35)	Design & Development	VI	0	1	0	0	0	0	0.08	Low

#### 3.2.1 Use-case 1: additional control sites

Seven instances had the aim of increasing the number of inputs available to the patient, six of which may be considered to be conventional in design. Brenner et al. aptly outlines the use-case as such:

“The ability to combine both body-powered and externally powered components and control systems, affords clinical prosthetists a genuine chance to reproduce each degree of freedom that is in need of replacement”. Brenner et al. (24)

In all six conventional cases, the patient had a transhumeral amputation, aligning with the previously documented prescription trends presented by McFarland et al. (12). Childress et al. documented the earliest example of hybrid-power in academic literature in 1970, examining the performance outcomes of replacing the mechanical elbow of a conventional body-powered prosthesis with an electric elbow. The study aimed to determine the preferable control scheme between either a pull-switch or myoelectric control (23). Brenner et al. prescribed two conventional hybrid-power prostheses to patients C and D, both composed of a myoelectric hand with a body-powered elbow & lock (24). Bouwsema et al. conducted a study into the differences in movement characteristics between hybrid-power and myoelectric prostheses, in which the three hybrid-power prostheses were all composed of body-powered elbows combined with myoelectric hands (25). Ricardo et al. presented a unique case in which a patient with an osseointegrated transhumeral amputation was assigned a hybrid-power prosthesis, utilising an electric elbow joint and body-powered terminal device (26). Thyberg et al. presented a case report utilising a myoelectric hand with a passive elbow and body-powered elbow lock (28).

Law et al. is the only novel clinical case with the goal of increasing the number of control sites. They noted that the patient was especially limited in their movement, only able to actuate one cable of their harness, eliminating the ability to both flex and lock the elbow using body-powered means. Instead of resigning to a passive lock mechanism, which the authors lament detracts from any functional improvement of the prosthesis, the authors constructed their own myoelectric shoulder lock (27). This intervention is similar to the MELA elbow lock investigated by Cupo et al. (53), but differs in that Law et al. provided active control rather than switch control. In practice, switch-controlled joints share more in common with a passive joint in a cosmetic prosthesis, requiring the use of an external entity such as an opposing hand to initiate the desired action (54).

#### 3.2.2 Use-case 2: physical effort reduction

The most salient trend in hybrid-power designs within a research context is reducing the physical effort required to operate the prosthesis. This concept is exemplified by Brenner et al., Spaeth et al.*,* Piazza et al., and Polhemus et al. (24, 29, 30, 33), who outsourced the physical effort to electric motors. The first example in literature emerged in 1981 with a case report from Spaeth et al. (29), in which a paediatric patient struggled to operate her transhumeral body-powered prosthesis due to the high input force required to operate the terminal device, a problem which body-powered prostheses still contend with today (55). Initially the clinician prescribed a myoelectric solution, however the patient was unable to produce signals of sufficient amplitude to operate the hand reliably. Instead, a cable-operated pull-switch was implemented which allowed the patient to operate the electric hand.

**Figure 5:**
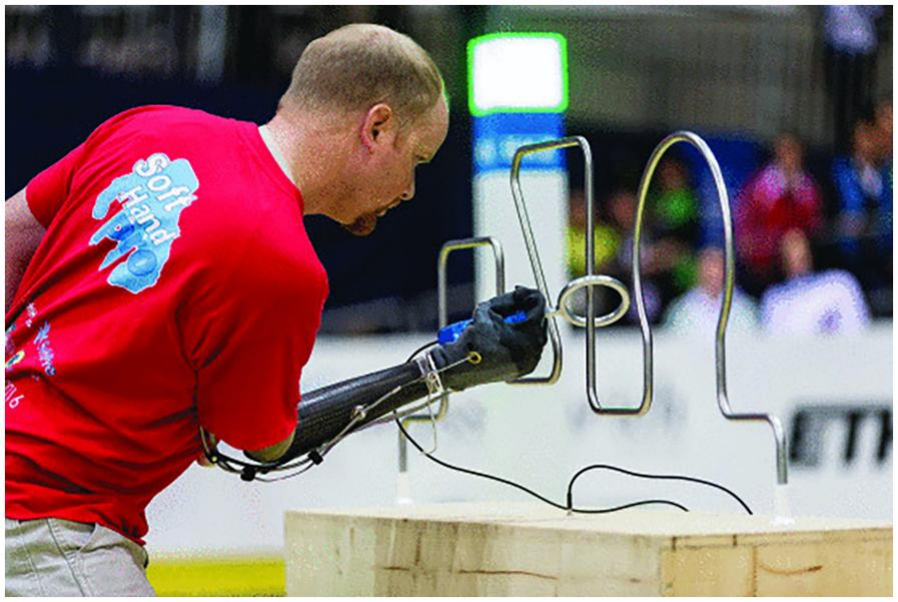
Pilot A operating the SoftHand Pro-H to perform the wire-loop task at the 2016 cybathlon competition (30). Photo used with permission of copyright holder, ETH Zurich.

Brenner et al. implemented a similar solution in 2008, with a transradial patient whose injury left him with limited use of his elbow and a lift capacity of only 4 pounds (1.81 kg), significantly limiting his ability to operate a body-powered terminal device (24). The patient was initially fitted with a cable-operated pull-switch system, which was later replaced with an electronic linear actuator, providing proportional rather than binary control. The third documented case is from Piazza et al. (30), showcasing a modification of the SoftHand-Pro, an underactuated electric hand concept first catalogued by Catalano et al. (56). This iteration, pictured in Figure 5, replaces myoelectric control with the cable-operated system by utilising a small lever in the hand that exerts proportional control over the finger movements. Godfrey et al. explored the potential of the SoftHand Pro-H for use in work environments, where myoelectric control may become unreliable due to the presence of sweat and dirt, by relocating all active components to an external compartment on the patient's waist (31).

Polhemus et al. proposed the uGrip II, a pneumatic hand and wrist concept which translates cabled harness movements into electrically-enabled pneumatic movements with the goal of minimising strain on the user (33). These joints are controlled using “five distinct movements” from a harness system to trigger a series of pull switches, with various state combinations dictating the wrist and finger movements. Conventional body-powered prostheses are limited to two harness-driven inputs, these being glenohumeral flexion and scapular protraction to engage the control cable, and shoulder depression with glenohumeral extension and abduction to trigger the elbow lock (29). How three additional movements may be introduced and reliably performed by the user is unspecified.

Seo et al. presented a partial-hand prosthesis, that measures the angular displacement of one intact digit on the back of the hand, which is translated into a matching degree of displacement in the fingers of absent digits, using a series of servomotors and linkages (57). The design is similar in principle to traditional partial-hand body-powered prostheses activated via the flexion of the wrist, with the benefit of leaving the wrist uncoupled from the hand which grants comparatively more freedom of movement.

#### 3.2.3 Other use-cases

**Figure 6:**
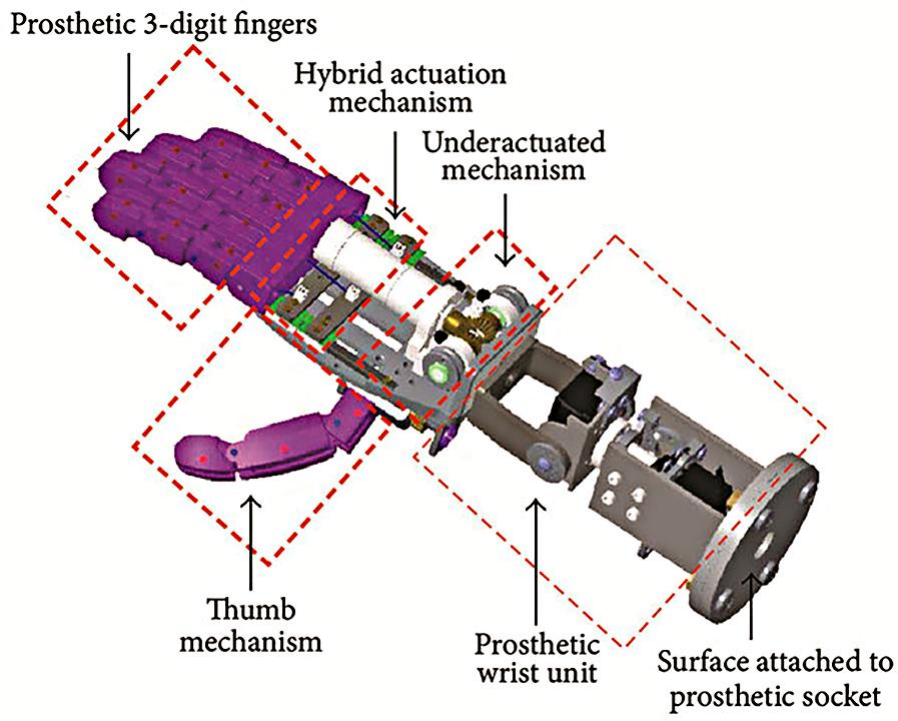
Hypro transradial hybrid-power prosthesis, designed by Semasinghe et al. (34).

Semasinghe et al. presents the HyPro, a hybrid-powered transradial device that exploits body-power and electric-power on the same joints, to increase the number of grasp types available to the user (34). Electric motors pre-position the fingers and thumb into one of five grasp patterns, whilst input from a body-powered cable performs the grasping actions by flexing the fingers. Such a design exploits the flexibility often associated with myoelectric prostheses, paired with the proprioceptive nature and finer control that is typically the domain of BP prostheses. The design itself is presented in Figure 6.

Reboli et al. proposed a transhumeral hybrid-power prosthesis that facilitates body-power *or* electric-power, for use in low-income countries where sourcing particular components for one or the other may be difficult (35). The terminal device operates using a simple linkage mechanism, which may be powered using either an internal linear actuator, or externally powered via a traditional cable and harness system. Zuniga et al. presented a transradial hybrid-power prosthesis with both body-power and myoelectric control over the fingers and thumb of the hand, designed for paediatric users to prevent muscular fatigue and scoliosis (58). Each control method can be exploited independently over the same joints, with the stated purpose being to reduce the strain typically associated with body-powered prostheses whilst encouraging users to exploit the strength they have where possible to develop the necessary muscle groups. The author comments that weaker motors may be intentionally implemented depending on the abilities of the patient to further encourage muscle development.

### 3.3 Performance outcomes of hybrid-power prostheses

Most clinical case reports of conventional hybrid-powered prostheses used patient-feedback as their outcome measure, which whilst essential for clinical prescription and patient outcomes, limits the amount of comparable qualitative data.

#### 3.3.1 Performance outcomes: additional control sites

Childress et al. conducted a series of timed trials across three participants with transhumeral level limb absence, where the performance of an electric elbow is investigated and compared between two control schemes: a pull switch and myoelectric signals (23). All three participants exhibited equal or better functional test scores using the externally powered elbow than when using the conventional body-powered elbow at the end of testing. The myoelectric control yielded faster test completion times than the pull switch in all participants by the end of training, which was reflected in each participants responses when asked of their preferred control scheme.

Bouwsema et al. performed a comparative study between myoelectric and hybrid-power prostheses, analysing their respective velocity profiles in a series of three grasping tasks (25). Two groups, each of three members were formed, a myoelectric group being all female with transradial amputations, and a hybrid group being all male with transhumeral amputations. The hybrid-power prostheses exhibited shorter movement completion times that exhibited more jagged trajectories than their myoelectric counterparts, suggesting less fluid and controlled movement. During direct grasping tasks, each hybrid-power prosthesis exhibited significant interruptions in their velocity profiles due to the uncoupling of the elbow joint, unlike the myoelectric prostheses which experienced higher velocity peaks.

Ricardo et al. monitored patient's progression over the course of 7 years, which was reported using the DASH questionnaire, 400 points test, and VAS (Visual Analogue Scale) (26). Measurements were taken with a traditional prosthesis pre-osseointegration, a hybrid-power prosthesis post-osseointegration, and the same hybrid-power prosthesis 7 years post-discharge. At each milestone, the patient exhibited significant improvements in the 400-points test performance, achieving 30%, 48%, and 69% at each respective milestone. The patient displayed a reversal in prosthesis use at work and home during this interval, with work use increasing from 25% to 80% and home use decreasing from 75% to 20%. This suggests an improvement in functional utility, but a preference to perform leisure activities without an assistive aid where possible.

Of the two patients prescribed conventional hybrid-power prostheses by Brenner et al.*,* both eventually exchanged their respective body-powered elbows for electronic switch-operated variants. Patient C was unable to flex her body-powered elbow more than 55 degrees due to her very short residual limb, and patient D likewise struggled flexing the elbow due to the weight of the electric hand and battery.

Thyberg et al. reported that following an extensive two-week training period, the patient used the prosthesis an average of 16 h daily at both the 6- and 12-month mark post-prescription (28). Grip function tests revealed the patient achieved scores within the lower range of transradial amputees, despite his transhumeral amputation, and performed similarly well across ten individual ADLs (activities of daily living). Law et al. presented no testing or outcome measurements for their proposed design (27).

#### 3.3.2 Performance outcomes: physical effort reduction

The SoftHand Pro-H (SHPH) measured it's success by its pilot's performance in the 2016 Cybathlon competition, a competition in which those with upper-limb amputations compete in a series of 6 tasks, aiming for the quickest time with the fewest number of errors. The pilot chose the hybrid over the myoelectric alternative, being used to a body-powered prosthesis in everyday life, and finished 2nd in the competition against 7 competitors. Functional tests showed the SHPH required 14 times less force to open to its maximum extent than a standard Ottobock hook, whilst achieving similar output grasping forces (30). The authors noted the biggest trade-offs were the loss of proprioceptive feedback and the weight of the solution, being heavier than the other devices trialled by 82%. A future study by Godfrey et al. addressed this by moving the motor, batteries, and PCB to the participant's waist (31). The authors compared this configuration against the previous compact design, and a traditional myoelectric setup version of the SoftHand Pro (59). Among the limb-intact subjects, the compact configuration slightly outperformed the waist-based design but not to any significant degree. The three participants with transradial limb absence all had no or low experience with body-powered prostheses whilst having medium or high experience with myoelectric prostheses. They all managed to perform the ACMC to a “generally capable” level with all three devices, with the myoelectric, compact and waist-based devices receiving scores of 55, 53 and 49 respectively. The difference in performance between the myoelectric benchmark and compact hybrid-power configuration were not statistically significant, suggesting equivalent function. The final investigation involving the SoftHand Pro-H was presented by Capsi-Moralles et al. (32), who recruited two pilots to participate in the 2020 Cybathlon Competition. In a test between the Softhand myoelectric and hybrid-power control scheme, the pilots achieved a greater SUS score than the hybrid, despite ACMC results indicating functionally similar performance. As a result, each chose the myoelectric device over the hybrid-power device to compete with.

Spaeth et al. discussed the fitting process of a 12-year-old paediatric patient, who initially struggled to actuate her body-powered prosthesis. With the simple cable-controlled electric hand prosthesis discussed, the author reported the patient being able to operate it “*without error*” following a 40 min training period, and the patient continued to express satisfaction with the device following a 12-month check-up (29).

Polhemus et al. present no testing or outcome measurements (33).

#### 3.3.3 Performance outcomes: other use-cases

Semasinghe et al. presented in-depth functional measurements including a kinematic evaluation and grasp analyses (34). Functional measurements by Semasinghe et al. found that the HyPro required 3.3 N of force to commence a chosen grasp, and that 1 N of body-powered force was required to exert every incremental 0.1 N of force from the fingertips. This indicates significant frictional losses which limit the HyPro's use in scenarios involving high loads (34). To create a 15 N pinching force, the user would need to exert 117 N of cable-force, far more than the forces required to operate a prosthesis fatigue free, and greater than most commercial body-powered terminal devices, as measured by Hichert et al. (55). Reboli et al. presented only minor functional measurements in the delay of motor and circuit signals (35).

## 4 Discussion

The primary aim of this review was to assess the state of research and evidence present in the ambiguous area of hybrid-power prostheses. To this end, the review aimed to categorise the disparate kinds of hybrid-prosthesis, to identify the use-cases of hybrid-power prostheses, and to determine the performance outcomes of said hybrid-power embodiments where possible. From this, the gaps in the literature could be identified, and subsequent research priorities and directions for future developments could be made. Thirteen studies and two patents examining the design and performance of various hybrid-power prostheses have been presented. A broad range of hybrid-power prosthesis designs were found, ranging in limb-difference level from partial-hand (57) to transhumeral (24).

### 4.1 Hybrid categories

The use of the term “hybrid” was found to be attributable to a variety of subcategories within prosthetic literature. It is important to note that these terms may not be exhaustive, but representative of the categories in which the articles captured in this review fell into. Despite this large variety, the number of studies found pertaining to hybrid-power prostheses specifically was low at only nineteen articles, of which only thirteen passed the full-text review criteria. Studies on hybrid-power prostheses were outnumbered by hybrid-control and hybrid-strategy prostheses, despite the former being the default definition for “hybrid-prosthesis”. The lack of subcategorization within this group means that whilst one hybrid group may be implemented by clinicians to allow patients struggling with traditional means of control to operate a prosthesis (hybrid-power), another may be implemented by programmers as a means of better interpreting sEMG data (hybrid-strategy). The clear variation within this nebulous group highlights the need for clearer and more descriptive categories to reduce ambiguity and misclassification. The author hopes the use of the suggested hybrid sub-categories within this review will help elucidate the wide range of prosthesis design and implementation techniques that falls under the descriptor of “hybrid” and encourage further development within the respective categories.

### 4.2 Hybrid-power use-cases

The primary goal of hybrid-power prostheses was found to be increasing the number of control sites available to the patient, such that they could exert active control over both the terminal device and elbow (23–26). Whilst in a research context, externally-powered prostheses often make use of multi-channel sEMG sensors in conjunction with classification algorithms to identify many potential output signals, commercial sEMG systems are typically limited to bipolar control with only two inputs to make use of. This is typically expressed by identifying suitable agonist/antagonist muscle pairs and using them to control opposing motions of the same degree of freedom. As such, the clinician's options to increase control inputs are significantly more limited than the state-of-the-art, and so combining the control potential of both body-power and external-power make a suitable compromise.

The next most common objective was the reduction of input force necessary to operate the terminal device, which led to largely positive outcomes for those the intervention was administered to (24, 29, 30). These interventions appear particularly useful for patients with short residual limbs, and those with other limitations in mobility that limit their ability to operate conventional prosthetic joints. The lack of evidence for their wider use in a clinical context may be attributable to the fact that when a patient is unable to operate a body-powered prosthesis effectively, the natural solution is to prescribe them a myoelectric solution instead. Only when the patient is also unable to effectively control the myoelectric solution, is a hybrid-power solution considered. This may be because hybrid-power solutions are more difficult to implement effectively than the other active classes, requiring knowledge by the prosthetist outside of typical clinical guidelines. This approach is limited to the scope of what can be achieved by combining disparate, prebuilt components. Hence, a larger variety of use-cases are observed in research contexts, where the researchers have the freedom to combine the active classes from inception, enabling more sophisticated and directed embodiments to arise. Such approaches are exemplified by Semasinghe et al. (34) and Seo et al. (57), both of whom present completely unique use-cases.

### 4.3 Hybrid-power performance outcomes

There was a notable lack of high-quality studies assessing the use and performance of hybrid-power prostheses. Most of the evidence on conventional hybrid-power prostheses are presented as case-studies, relying solely on qualitative evidence from patients. Of the three studies that employed the highest level of evidence present in the captured studies, that being cross-sectional design, only two investigated what could be considered a *conventional* hybrid-power design. Bouwsema et al. (25) presents a direct comparison on the kinematic profiles between a conventional hybrid-power and electric prostheses, however the comparative analysis is severely limited by each group having a different level of upper-limb absence. Only the study by Childress et al. (23) assessed the functional performance of a conventional prosthesis with respect to a control, Whilst Godfrey et al. presents additional insight by revealing equivalent performance between a novel hybrid-power configuration and a similar myoelectric configuration, it does not provide much actionable data for current clinical practice (59). All three participants with upper limb difference had much more experience with myoelectric prostheses than body-power prostheses and were able to achieve equivalent performance following a brief training period. This suggests an ease of learning and use in similar hybrid-power architectures, which could be of use to paediatric users when introducing them to active prostheses. This aligns with Zuniga et al. proposal of a training prosthesis, by combining the intuitive control of a harness-based system with the low physical strain of an electric system (58). The subsequent study performed by Capsi-Morales et al. had both documented Pilot B, a cosmetic prosthesis user with previous myoelectric experience, achieve slightly higher ACMC using the hybrid-power configuration (BC-c1) over the myoelectric solution, yet rate the former substantially lower in the SUS (32). This could suggest a familiarity bias towards the myoelectric configuration, or a more considerable difference in the user experience not captured by the presented data.

Childress et al. present insight into the performance of a transhumeral hybrid-powered prosthesis compared to a conventional body-powered prosthesis, suggesting a functional improvement in all participants when cable control of the elbow is replaced with myoelectric control. Of the studies discussed in this review, there was only one overlap in comparative outcome measures, namely the ACMC and SUS. However, these were for the same instance across different studies. The confidence ratings of the respective papers were somewhat low, with only two papers achieving a confidence rating greater than 0.7. As such, it is difficult to make any substantiated claims regarding the functional performance of hybrid-power prostheses. It is clear that there is a substantial gap in research on the functional performance of conventional hybrid-power prostheses. This scoping review is not the first to reach this conclusion, with other researchers like Piazza et al. advocating for similar interventions:

“Despite the fact that solutions combining BP harnesses, linear transducers, and electrically powered hands are possible, there is limited use of such systems and a lack of literature investigating such hybrid setups”—Piazza et al. (30)

As previously discussed, body-powered prostheses are the dominant choice of active prosthesis for users wishing to pursue work involving manual labour, due to their perceived robustness and reliability. The work of Godfrey et al. highlights an opportunity to introduce the versatility of external-power to work prostheses without compromising their robust nature, as exemplified by their proposed “hardy” configuration (31). Hybrid-power may be used as a means of introducing greater variety to the functions of prosthesis-users in manual-labour roles, outside of what is typically limited to opening/closing of the terminal device and flexing of the elbow.

### 4.4 Implications for clinical practice

The work presented has made clear that whilst hybrid-power prostheses have received positive patient feedback when prescribed in clinical practice, there is insufficient evidence to provide any substantiated guidelines regarding their implementation. Nevertheless, several conclusions may still be drawn from the findings of this review based on trends identified across clinical case-studies and opinion-pieces. These conclusions are put forward for the consideration of clinicians interested in integrating hybrid-power prostheses into their prescription toolkit, as a means of assessing whether a patient may benefit from a less traditional intervention.

1. All captured clinical cases of hybrid-power architecture have been prescribed to patients with trans-humeral limb absence (24, 26, 28, 29). Of these prescriptions, all hybrid-power devices utilised an electric terminal device with a body-powered elbow or vice versa, usually with additional passive components.
2. Hybrid-power designs were usually considered in cases where patients had previously tried and struggled with a single active-class prosthesis. This may arise from lacking the physical strength or mobility to operate several body-powered components (24, 26, 28), or the control necessary to precisely operate a myoelectric intervention (29).

When determining the optimal prescription for a patient, the clinician may consider making a decision not on the class-level, but on the *component-level*. Whilst many, if not a majority of patients may benefit from just a single-class prosthesis, some patients may have more nuanced needs and capabilities that may lend themselves to a hybridised solution. For instance, consider a patient that with trans-humeral limb-absence, who desires the heightened proprioception associated with body-powered solutions, but lacks the strength to operate multiple cable-driven joints. When considered on a class-level, the only feasible option may be a myoelectric prosthesis, using an electric elbow and hand. However, when considered on a component-level, the patient may still benefit from the electric elbow to manage lifting forces, but could make use of a low-force cable-operated split hook such that proprioception is retained. Whilst this consideration may be obvious to clinicians well-acquainted with hybrid-power design structures, it may serve as a useful framework for clinicians open to incorporating more novel solutions into their workflow.

### 4.5 Future work

Whilst the literature presented in this review presents a promising picture for the use of hybrid-power prostheses, the documented cases are too few and varied to draw any substantive conclusions on the functional performance of hybrid-power prostheses relative to other active prosthesis classes. To fill this gap, the authors recommend a thorough study into hybrid-power prostheses with the most representative population, that being a patient with trans-humeral limb absence. Whilst this has previously been explored by Childress et al., the study stands at over half a century old at time of writing, during which time electric prostheses and robust outcome measures have evolved significantly. Such a study would ideally explore four unique prosthesis variations:

1. **Body-power**; Body-powered elbow, body-powered terminal device
2. **External-power**; Electric elbow, electric terminal device
3. **Hybrid 1**; Body-powered elbow, electric terminal device
4. **Hybrid 2**; Electric elbow, body-powered terminal device

This comparison would provide adequate controls against which to compare the hybrid-power designs and identify the functional differences when swapping the respective component classes. Functional outcome measures could include the SHAP test (Southampton Hand Assessment Procedure) (60), BBT (61), ACMC (62), or SUS (63). In addition to functional capacity, the measurement of patient cognitive load during such activities would be greatly beneficial, to determine if the use of two disparate control paradigms simultaneously has a significant impact on performance. Such a study may go a long way to establishing hybrid-power architectures as a mainstay in the toolset of clinicians.

Outside of a clinical context, the hybrid-power designs emerging from research have shown that the breadth of use-cases for hybrid-power architectures is far greater when the classes are designed together from inception. Considering one of the core use-cases for hybrid-power prostheses is the introduction of more control-sites, it is surprising that the hybrid-power architecture has not been exploited to expand the degrees of freedom available for transradial prosthesis users. Whilst Semasinghe et al. primarily explored the use of hybrid-power to introduce multiple grip types to a bionic hand, few efforts have been made to use hybrid-power as a means of reliable active wrist control. Only two studies in this review propose an active wrist unit (33, 34), and neither test the control of such a design with an upper-limb prosthetic user. In four instances the authors opted for a passive rotation wrist unit, and in six instances the wrist was not reported on at all (24–26). The wrist unit seemingly being an afterthought appears to be part of a broader theme in upper-limb prosthetic development. In spite of its absence in prosthesis design proposals, many researchers have advocated for an increased focus on active wrist units as a key priority for both transradial and transhumeral prosthesis users (1, 64–66). This is unsurprising considering the significant role wrist movement plays in everyday object manipulation and acts of daily living (67–69). The case for hybrid-power wrist control is bolstered when considering the hybrid-power architectures' potential for simultaneous control of multiple joints (3), which is difficult to achieve with even sophisticated modern myoelectric prosthesis signal processing techniques. As such, the authors encourage researchers to explore this gap by considering the possibilities that trans-radial hybrid-power prostheses focused on active-wrist control may provide for users.

### 4.6 Limitations

The scope of this review is limited in its search criteria necessitating the use of the term “hybrid” to describe the prosthesis in question. This may exclude resources existing prior to the popularisation of the term, or articles by authors using terminology outside of the ambiguous standard for hybrid prostheses. In addition, the identification of the three new categories of “hybrid” was based solely on perceived topic trends during the screening phase of the review, and did not follow a strict methodical designation, and subject to the bias of the authors.

Additionally, only one author performed the data extraction from each of the articles following screening by two authors (ECP and SW), posing a potential for bias in the reporting of the presented articles. Of the articles discussed, the majority were deemed to be of low confidence, due to the lack of studies using participants with upper-limb amputations, and a lack of standardised or comparable outcome measures. The low number of studies explored in this paper, outside of the limited research within this area, is exacerbated by the exclusion of non-English language papers from this study. This is a consequence of the research team being predominantly native-English speakers with a lack of additional skills, limiting the scope of resources that could be explored within practical means.

## 5 Conclusions

Hybrid-power prostheses present a novel means of addressing difficulties in user control that are rarely explored in research, despite their establishment in clinical practice. This may perhaps be due to the ambiguity of the area, being home to five subcategories of “hybrid”, which have now been identified in this study. The thirteen research studies and two patents discussed show a wealth of potential and variation amongst embodiments of the hybrid-power format, however, the majority appear to fall into one of two use-categories: increasing the number of control sites available to the patient, and reducing the physical effort expenditure of the patient during prosthesis use. Whilst the presented case-studies indicate satisfactory user function when using conventional hybrid-powered devices, there is very little quantitative evidence regarding their functional outcomes that could be used to assess them against their active counterparts. To fill this research gap, it is strongly recommended that researchers use standardised clinical means to establish a foundation of knowledge on these hybrid-power devices, such that future innovations on hybrid-power devices may draw upon reliable findings.

## Data Availability

The original contributions presented in the study are included in the article/Supplementary Material, further inquiries can be directed to the corresponding author.
